# Measuring Language Teacher Emotion Regulation: Development and Validation of the Language Teacher Emotion Regulation Inventory at Workplace (LTERI)

**DOI:** 10.3389/fpsyg.2021.708888

**Published:** 2021-08-23

**Authors:** Tahereh Heydarnejad, Gholamreza Zareian, Saeed Ghaniabadi, Seyyed Mohammad Reza Adel

**Affiliations:** Department of English Language, Faculty of Literature and Humanities, Hakim Sabzevari University, Sabzevar, Iran

**Keywords:** emotion regulation strategies at workplace, EFL teachers, the language teacher emotion regulation inventory, instrument validation, the educational psychology

## Abstract

Educational context is a pool of various emotional demands asking for competent teachers who are capable enough to regulate and manage them. The language teacher emotion regulation focuses on the strategies that language teachers implement to regulate their emotions. Considering the paucity of a psychometrically sound instrument in language teacher emotion regulation, this realm has received scant research attention. Thus, the present study was an attempt to develop and validate a conceptually meaningful and psychometrically sound instrument to capture language teacher emotion regulation strategies at workplace. This study is composed of three phases. In the first phase, based on a comprehensive consideration of the existing literature and the results of a semi-structured interview, a six-component model of the language teacher emotion regulation was designed. In the second phase, the results of exploratory factor analysis (EFA), confirmatory factor analysis (CFA), and reliability estimates confirmed the validity and reliability of the instrument. The results of CFA refined the final version of the instrument. The Language Teacher Emotion Regulation Inventory (LTERI) includes 27 items with six dimensions on a 5-point Likert scale. Each dimension assesses a discrete language teacher emotion regulation strategy at workplace: situation selection, situation modification, attention deployment, reappraisal, suppression, and seeking social support. In the third phase, the validated instrument, LTERI was utilized across two different milieus of language teaching in Iran, namely school and university. To do so, an independent samples *t*-test was applied. As the findings of this phase demonstrated, there is a statistically significant difference between English as a foreign language (EFL) teachers in the two contexts regarding the employed emotion regulation strategies in their professional lives. The implications of the current study can open new perspectives in educational psychology and teacher well-being. Furthermore, the Language Teacher Emotion Regulation Inventory (LTERI) contributes to the field of teacher education by filling the measurement lacuna and advancing quantitative studies in this regard. More significantly, the implications of this study may uncover new prospects for effective teaching and learning, especially during the COVID-19 pandemic, which can provoke various emotional demands for both teachers and learners.

## Introduction

Teachers are continually exposed to a wide variety of pleasant and unpleasant emotional experiences at work. On the intense wave of emotional experiences, teachers are expected to be competent to manage and manipulate their emotional demands because the way teachers interpret their emotions will affect their decision, instruction, and well-being. They need to modify the intensity and duration of the emotional experiences at work, which can be viewed through the lens of emotion regulation (Chang and Taxer, 2020). Thus, emotion regulation is a seminal constituent of teachers influencing teachers, learners, colleagues, parents, educational context, and consequently the whole society.

Emotion regulation is a complex process that includes launching, hindering, or adapting individuals' state or behavior in a given situation (Gross, 1998). That is, emotion regulation affects the processes through which teachers modify their emotions (Gross, 2014). Teaching includes both mastering how to communicate subject matter to the learners and managing the emotional dimensions of education (Richards, 2020). In other words, the more teachers regulate their emotions, the more they are able to employ their mental faculties and in consequence, effective teaching is guaranteed (Alipour et al., 2021; Heydarnejad et al., 2021). More specifically, the attachment of rationality with emotionality as the two wings of teachers leads to efficient teaching (Chen and Cheng, 2021).

Language teaching is an emotionally-charged activity (Richards, 2020). In language teaching, teachers should teach language and culture simultaneously, which can trigger various challenges and emotion-provoking stimuli (Tsang and Jiang, 2018). Emotional regulation acts as a modifier; it helps language teachers modulating responses triggered by emotional demands. To achieve emotion regulation goals, different strategies are used: self-awareness and self-regulation (Heydarnejad et al., 2021), reappraisal and adaptability (Burić et al., 2017), as well as suppression (Chang and Taxer, 2020), to name a few. Existing literature on teacher emotion regulation viewed emotion regulation through the lens of emotional labor (Philipp and Schüpbach, 2010) or suppression and cognitive reappraisal (Chang, 2013).

Although prior research has provided valuable insight into how regulating emotions influence effective teaching, it is limited and entirely in its infancy, particularly in the domain of language teachers. To date, few quantitative research has been done on language teacher emotion regulation and this lacuna may be due to the lack of a psychometrically multidimensional self-report instrument to measure strategies that language teachers implement to regulate their experienced emotions at work. To fill this gap, the current study intended to come up with a model of the language teacher emotion regulation and to design an instrument to allow for the quantification of the construct and its empirical investigation, respectively. In addition, having determined the validity and reliability estimates of Language Teacher Emotion Regulation Inventory (LTERI), the possible difference between EFL teachers' emotion regulation strategies in two different contexts (high school and university) was studied.

## Literature Review

### Emotion Regulation

The word emotion is originated from the Latin word “emovere” meaning to stimulate (Hargreaves, 1998). That is, individuals are driven by their experienced emotions. Emotions are multi-componential phenomena (Shuman and Scherer, 2015); they include several paralleled psychological processes. Emotions consist of affective, cognitive, physiological, motivational, and expressive constituents (Burić et al., 2017). Zembylas (2004) considered emotions as relational, evaluative, and political launching by the politics and power relations within an educational system and the whole of society. In the same vein, Izard (2010) viewed emotions as involving cognitive appraisal, cognitive interpretation, neural systems, and expressive behavior. Emotions are not fixed and can be managed and regulated to match experienced situations.

The teaching profession is bounded with different experienced emotions, which affect teachers' cognitions (Sutton and Wheatley, 2003; Sutton, 2004), motivation (Pekrun et al., 2002), efficacy beliefs and goals (Kaplan et al., 2002; Chen, 2018), memory, attention, and categorization (Sutton and Wheatley, 2003), self-regulation (Heydarnejad et al., 2017), immunity and autonomy (Azari Noughabi et al., 2020), pedagogical adoptions (Chen, 2020), forming a sense of professional identity (Day and Qing, 2009), self-efficacy (Burić et al., 2020), social well-being (Richards, 2020), teaching style (Heydarnejad et al., 2021), and consequently their students' learning and achievement (Frenzel, 2014). Among different emotions that teachers experience at work, they are expected to express pleasant emotions such as happiness, joy, and pride but down-regulate unpleasant emotions such as anger, frustration, and anxiety (Schaubroeck and Jones, 2000). In a pool of daily experienced emotions, teachers as the center of the class are supposed to avoid expressing too strong and too weak emotions (Chen, 2020; Chen and Cheng, 2021). To this end, teachers' emotion regulation strategies are fundamental which can promote their professional well-being and growth.

Emotion regulation refers to spontaneous or controlled processes adopted to control and manage positive and negative emotional experiences (Gross and Thompson, 2007). That is to say, emotion regulation is the process, which gives meaning and direction to individuals' emotions (Gross, 1998). Emotions are multi-component procedures and emotion regulation can alter the latency of emotional responses, their rises, and duration besides their cognitive, behavioral, and physiological reactions (Sutton, 2004; Burić et al., 2017; Taxer and Gross, 2018). Among the concepts related to the management of emotional experiences, emotion regulation, emotional labor, and coping are sometimes mixed, although they are different. Emotional labor is a concept coined by Hochschild (1983), referring to the management of individuals' emotions to act according to the commands of occupational norms (Choi and Kim, 2015). That is, emotions are adjusted to organizational expectations. In this regard, two forms of emotional-labor strategies are described (Hochschild, 1983): surface acting and deep acting. Surface acting concerns stimulating emotions that are not experienced to reach the desired emotion. Deep acting is the adjustment of inner feelings to express organizationally desired emotion. Thus, in surface acting, feelings are changed from outside in, while in deep acting, feelings are changed from the inside out (Hochschild, 1983). Emotion regulation involves the management of both pleasant and unpleasant emotions but coping is considered as cognitive and behavioral attempts to master, reduce, or tolerate stress (Klapproth et al., 2020). Two main coping strategies are defined for stress management: problem-focused and emotion-focused. Problem-focused coping strategies appertain to the efforts taken to alter the source of the stress. In contrast, emotion-focused coping strategies address the attempts to change individuals' emotional responses to the stressor (Folkman and Lazarus, 1985).

Research in the domain of teacher emotion regulation is quite rare. A glance into the existing literature on teachers' emotion regulation mirrors studies, which are restricted to negative emotions within a framework of coping and stress (Lewis, 1999), emotional labor (Isenbarger and Zembylas, 2006), or self-regulation (Fried, 2011). In a qualitative study by Jiang et al. (2016), the results of students' surveys and teachers' interviews indicated that reappraisal is more effective than suppression in increasing positive-emotion expressions and reducing negative-emotion expressions. In a recent study by Chang and Taxer (2020), teacher emotion regulation strategies in response to classroom misbehavior were explored. Based on their findings, teachers who usually reappraise are less likely to have unpleasant emotional experiences in the face of students' misbehavior and express fewer suppression experiences when negative emotions are felt. By the same token, Chang (2020) explored the association between teachers' beliefs about emotional display rules in the class, the attitudes toward emotion regulation strategies, and feelings of burnout. The results of this study demonstrate that display rules are very influential in expressive suppression and burnout. Moreover, cognitive reappraisals negatively correlated with teacher burnout.

### Theoretical Framework of the Study

To explain emotion regulation, different models have been generated. For instance, the Hot/Cool System of emotion regulation stimulates regulation into willpower (Mischel and Ayduk, 2004). The cool system empowers people to remain calm when they experience intensive emotional disturbances; on the other hand, the hot system works as a quick emotional processing (Mischel and Ayduk, 2004). The hot system develops in the first stages of life, but the cool system emerges in adulthood. Based on this model, teachers who can manage their emotions successfully develop a cool mechanism by ignoring the stimulus, distracting themselves, or enclosing another meaning to the stimulus (Sutton and Harper, 2009). Resources or Strength Model is another emotion regulation model (Schmeichel and Baumeister, 2004). The idea behind this model is rooted in self-regulation in general and emotion regulation in particular. Also, the role of motivation in successful emotion regulation is highlighted in this model (Muraven and Slessareva, 2003).

Among all, the process-oriented model of emotion regulation proposed by Gross (1998) is a comprehensive model with five temporal points. Situation selection as the first component of emotion regulation process model implies actions that individuals apply to end situations that will cause a particular emotion. Situation modification, the second component, refers to the processes employed to change the qualities of situations that provoke specific emotions. Attention deployment as the third component of emotion regulation refers to the redirection of individuals' attention to affect individuals' emotions. The cognitive change includes strategies adapted to alter the cognitive appraisal of a situation that evokes emotional experiences. The final component of the emotion regulation process is called response modulation, referring to different strategies used to magnify, dwindle, or lengthen the physical, experiential, or behavioral reactions. These five processes indicate five families of emotion regulation processes. In this model, the first four processes are anticipate-focused due to their usage before complete activation of the emotional responses, whereas the last component is utilized to modulate the consequences of fully developed emotional responses (Gross and Thompson, 2007).

As it was mentioned before, research in the domain of teacher emotion regulation is still in its infancy and calls for more attention (Jiang et al., 2016; Burić et al., 2017; Taxer and Gross, 2018; Chang, 2020; Chen and Cheng, 2021). Moreover, not enough is yet explored about the contextual triggers and components of language teacher emotion regulation (Richards, 2020; Alipour et al., 2021). In addition, prior research has missed to quantitatively explore facets of language teacher emotion regulation. This gap may be due to limited attention to language teacher emotion regulation as well as lack of a valid and reliable measurement of language teacher emotion regulation. The existing instrument to measure teachers' emotion regulation is the “Emotion Regulation Questionnaire” by Gross and John (2003) and “Teacher Emotion Regulation Scale” (TERS) by Burić et al. (2017). “Emotion Regulation Questionnaire” consists of 10 items that measure individual eagerness and inclination to manage their emotions in two aspects: Cognitive Reappraisal and Expressive Suppression. This scale is general and not particularly devoted to the teaching context. In addition, it just explores emotion regulation from two broad perspectives: cognitive reappraisal and expressive suppression. The other instrument, the “Teacher Emotion Regulation Scale” (Burić et al., 2017), is a precise and detailed measurement including five dimensions: Avoiding the Situation, Active Modification Strategy, Reappraisal, Suppression, and Tension Reduction. However, this instrument is generally suggested in teaching context, but emotion regulation is context-bound (Gross, 1998). In particular, language teaching is closely tied to various emotional experiences (Khajavy et al., 2018); it is a matter of socialization (Richards, 2020). Furthermore, the existing instruments do not reflect the dimension of seeking social support, which is closely connected with the act of teaching (Jennings and Greenberg, 2009; Taxer and Gross, 2018). To fill the gaps, the present study aimed at developing a psychometrically sound multidimensional self-report instrument to assess language teacher emotion regulation. To achieve these objectives, both theoretical conceptualization and empirical analysis were utilized. Henceforth, the following research questions are addressed in the current research:

1. What are the contextual triggers and components of language teacher emotion regulation?
2. Is Language Teacher Emotion Regulation Inventory (LTERI) a valid and reliable tool?
3. Is there any significant difference between EFL teachers' emotion regulation strategies across two educational contexts (high school and university)?

## Methodology

Three phases were conducted to address these research questions. In the current study, Dörnyei and Taguchi's (2010) guideline was utilized as the main source for questionnaire development and validation. A detailed account of the proposed methodology is given below.

### Phase 1

#### Designing the Scale

The first phase of the present study included different steps to explore language teachers' emotion regulation strategies and to design the initial item pools for the Language Teacher Emotion Regulation Inventory (LTERI). Based on a comprehensive review of the literature, the theoretical assumptions on the emotion regulation in general, and teacher emotion regulation in particular (Gross, 1998; Gross and John, 2003; Jennings and Greenberg, 2009; Burić et al., 2017; Taxer and Gross, 2018), a hypothesized model for language teacher emotion regulation was developed. This proposed model comprises six dimensions, including situation selection, situation modification, attention deployment, reappraisal, suppression, and seeking social support. In this model, situation selection, situation modification, and attention deployment are the dimensions suggested by Gross's the process-oriented model of emotion regulation (Gross, 1998), reappraisal and suppression are added based on Gross and John's study (Gross and John, 2003), and seeking social support is the last emotion regulation component in our proposed model inspired by Jennings and Greenberg (2009) as well as Taxer and Gross (2018). Next, an initial item pool was generated considering the theoretical assumptions of our proposed six-dimensional model of language teacher emotion regulation and adapting some items from the existing instruments (Burić et al., 2017). Among all the potential strategies used by teachers to regulate the emotions experienced across different situations, the purpose of this study is to include the emotion regulation strategies experienced at work. Moreover, to complement the conceptual relevance of this hypothesized model in the Iranian context, a semi-structured interview with 22 EFL high school teachers was conducted. After the verbatim transcription of the audiotaped data and thematic analysis, some of the items in the initial pool were discarded or revised. Then, the face and content validity of the instrument was explored.

#### Participants and Procedures

The participants of this phase were 22 Iranian teachers teaching English as a foreign language at state or private high schools in Iran. Their age varied from 25 to 50 years old with 1 to 27 years of experience. Among them, four teachers were Ph.D. candidates, 11 had an MA degree, and 7 held a BA degree. The majority had majored in English teaching, but four teachers had majored in English literature and three in Translation studies. Owing to the tight constraints on physical and social distancing caused by the COVID-19 pandemic and the convenience of on-line communication, on-line interviews were held, which could eliminate the geographical barriers. After the researcher's contact with the teachers and gaining their permission for the interview, semi-structured interviews were conducted individually with each participant. Furthermore, each participant was interviewed in one session and each session took about 30–40 min. The participants were interviewed on emotional experiences at work and the effects of such emotions on their behavior. They were also asked what they want/wish they can do then. Furthermore, the participants were asked to elaborate on emotion regulation techniques that they usually employ to manage their emotional experiences at workplace (see Appendix I).

After the verbatim transcription of the audiotaped data, thematic analysis (deductive approach) was conducted based on Braun and Clarke's guidelines (Braun and Clarke, 2006) and principles of qualitative data analysis (Ary et al., 2014). To do so, the researchers reviewed the transcript of every interview and highlighted all the phrases and sentences corresponding to relevant codes to teacher emotion regulation. Next, the codes were reviewed to identify the patterns among them and combining them into themes (i.e., situation selection, situation modification, attention deployment, reappraisal, suppression, and seeking social support). Based on the findings of thematic analysis as well as reviewing and grouping what they purportedly measured, some of the items in the initial item pool were discarded or revised. For instance, in the initial item pool, one item in the situation selection dimension suggests that a language teacher may cancel a class when he/she is annoyed by the students, but in semi-structured interviews no participants refer to this emotion regulation strategy. In another item in the initial item pool (situation modification stage), this strategy was suggested: I yell at students when they make me angry in language classes. None of the Iranian EFL high school teachers in their semi-structured interviews refer to this strategy. As Iranian EFL high school teachers said, they prefer to modify this situation positively. Thus, this item was revised as “If my students make me angry in language classes, I try to advise them.”

Furthermore, to ensure the content and face validity of the scale (Dörnyei and Taguchi, 2010), a group of experts (two educational psychologists, two psychometricians, two university teachers who majored in applied linguistics, and two English teachers) evaluated the quality of items in terms of clarity and comprehensiveness. Accommodating the experts' views and revisions resulted in a more refined and comprehensible version of the instrument. In addition, this instrument was administered to 60 participants similar to the target population, which finally resulted in 31 items (Table 1). Item piloting results in a more refined and comprehensive scale (Dörnyei and Taguchi, 2010).

**Table 1:** Emotion regulation dimensions in the proposed model with examples.

	**Dimension**	**Example**
1	Situation selection	I try to avoid discussing with troublesome parents.
2	Situation modification	If my students make me angry in language classes, I try to advise them.
3	Attention deployment	When I feel anxious in my language classes, I shift my attention to something pleasant.
4	Reappraisal	When I feel upset in my language classes, I redirect my attention to more pleasant matters.
5	Suppression	If I feel anxious in my language classes, I try to suppress that.
6	Seeking social support	When I feel frustrated in my language classes, I share my troubles with my colleagues.

### Phase 2

#### Exploring the Validity and the Reliability of the Scale

To check the test-retest reliability of the instrument, it was administered to 30 participants similar to the target population. Then, the instrument was administered 2 months apart to these 30 participants, and the correlation between the two sets of results was calculated. The results of the Spearman correlation were high (*rs* = 0.91, *p* < 0.05), showing very high test-retest reliability. Moreover, the internal consistency of the questionnaire was assessed through Cronbach's Alpha coefficient. Experts' judgments, as explained earlier, were employed to examine the content validity of LTERI. To explore the factor structure of LTERI (with 31 items) and to confirm the number of its underlying factors representing the dimensions, both exploratory factor analysis (EFA) and confirmatory factor analysis (CFA) were run following the methodological considerations by Meyers et al. (2016). Each developed component of the scale was considered as a latent variable demonstrated through a number of indicators (i.e., questionnaire items). To ascertain the final number of items in EFA, three criteria were utilized: (1) Kaiser-Meyer-Olkin Measure of Sampling Adequacy (KMO), (2) Bartlett's Test of Sphericity, and (3) Principal Components Factoring (PCF) with varimax rotation (Pallant, 2007). Furthermore, CFA checked the interrelationships among latent variables and whether its corresponding measured variables were valid or not.

#### Participants and Procedure

The required data for exploring the factor structure of LTERI through EFA and CFA would be collected by administering the scale to 420 EFL teachers with an age range between 23 and 53 (*M* = 35.967, *SD* = 9.290) from diverse geographical areas of Iran. Among 420 participants in this phase, 43% were male, 57% were female, and their teaching experience ranged from 1 to 30 years (*M* = 12.833, *SD* = 9.048). Out of 420 EFL school teachers, 21 teachers were Ph.D. candidates, 147 held an MA degree, and the rest had a BA degree in different branches of English studies: English teaching (214), English literature (107), Translation Studies (78), and English Linguistics (21).

Given that there is no clear consensus about the size of the sample for conducting factor analysis (Weston and Gore, 2006), the proposed number of participants is solely based on the assumption that the data for this study would not have any problem such as non-normal distribution or missing data. Google Forms was utilized to make electronic copies of the questionnaire and they were shared with the intended number of participants via email or Telegram application.

### Phase 3

#### Applying the Validated Scale across Two Educational Contexts

In the third phase of the study, the validated instrument was utilized to determine whether the validated scale of LTER functions equivalently among EFL high school and university teachers and whether there are any disparities in emotion regulation strategies between these two groups of teachers.

#### Participants and Procedure

A total number of 534 EFL high school teachers (258 male and 276 female) and 476 EFL university teachers (267 male and 209 female) took part in this phase. To ensure generalizability, it was attempted to include EFL high school and university teachers from different age groups with different teaching experiences from various provinces of Iran (Fars, Hamedan, Isfahan, Kerman, Khorasan Jonoubi, Khorasan Razavi, Khorasan Shomali, Qom, and Tehran, among others). A web-based platform was used to conduct the phase. Altogether, 1,010 forms were received with a 75% return rate, and no data were missed on account of the design of the electronic survey. Then due to the normality of the data, an independent-samples *t*-test was run to determine the possible difference between EFL high school and university teachers in preferred emotion regulation strategies.

The profile of the teachers is as follows:

High school teachers were between 23 and 53 years old (*M* = 38.100, *SD* = 9.125) with 1–30 years of teaching experience (*M* = 14.833, *SD* = 9.184). Out of 534 EFL high school teachers, 258 were male and 276 were female. Among them, 323 had majored in English teaching, 105 in English literature, 81 in Translation studies, and 25 in Linguistics. About their educational background, 33 teachers were Ph.D. candidates, 287 held an MA degree, and the rest had a BA degree.
University teachers' age varied from 27 to 52 years old (*M* = 36.767, *SD* = 6.907) with 1–25 years of teaching experience (*M* = 10.733, *SD* = 6.833). They were 267 male and 209 female. They had majored in different branches of English, i.e., English teaching, English literature, Translation studies, and Linguistics. Among them, 395 teachers were Ph.D. or Ph.D. candidates, and 81 had an MA degree.

## Results

### The Hypothesized Model and the Contextual Triggers of Language Teacher Emotion Regulation

Based on the review of the existing literature on emotion regulation, a semi-structured interview with 22 EFL high school teachers as well as consultations with experts, the contextual triggers, and the components of language teacher emotion regulation (LTER) were designed. Based on the results, the contextual triggers of language teacher emotion regulation were suggested as following: learners' misbehavior, unpleasant discussions, troublesome parents, disrespectful colleagues, and inattentive learners or laziness of learner to name a few. For instance, one teacher said, “I do not attend a meeting when I know my disrespectful colleague is there; I avoid being in such situations.” Another teacher refers to social support as a helpful and constructive strategy for regulating emotions. Iranian EFL high school teachers elaborated on the emotion regulation strategies that they prefer to employ at the workplace, which were thematically grouped as situation selection, situation modification, attention deployment, reappraisal, suppression, and seeking social support. Finally, LTER with six dimensions and 31 items was developed.

### Exploratory Factor Analysis (EFA)

To explore the latent structure of the LTERI, exploratory factor analysis (EFA) was employed. Firstly, as a measure against multicollinearity, Barlett's test of sphericity and the Kaiser-Meyer-Olkin (KMO) measure of sampling adequacy were used. According to Table 2, the KMO value was 0.859 exceeding the recommended value of 0.6 (Kaiser, 1974); therefore, the sample size for employing EFA was convenient. Also, the result of Bartlett's Test of Sphericity was significant (*p* < 0.05), indicating that the factor analysis is considered appropriate (Tabachnick and Fidell, 2007).

**Table 2:** KMO and Bartlett's test.

Kaiser-Meyer-Olkin measure of sampling adequacy		0.859
Bartlett's test of sphericity	Approx. Chi-square	11481.686
	df	465
	Sig.	0.000

Then, to empirically support the existence of separate factors for language teacher emotion regulation and test a theoretical model of latent factors, Principal Components Factoring (PCF) with varimax rotation was utilized. PCF with varimax rotation on the 31 items of the Fitting Dataset presented six sub-scales (Situation Selection, Situation Modification, Attention Deployment, Reappraisal, Suppression, and Seeking Social Support) with eigenvalues greater than one accounting for 57.093% of the total variance and 0.45 as the minimum item loading threshold was considered (Raubenheimer, 2004). Table 3 reported the results of exploratory factor analysis (items with a loading threshold of more than 0.45 were marked in bold).

**Table 3:** The results of exploratory factor analysis.

**Rotated component matrix^a^**						
	**Component**					
	**Situation selection**	**Situation modification**	**Attention deployment**	**Reappraisal**	**Suppression**	**Seeking social support**
q1	**0.64**	0.084	0.033	0.199	0.344	0.020
q2	**0.408**	0.218	0.092	0.218	0.016	0.167
q3	**0.58**	0.316	0.208	0.028	0.357	0.257
q4	**0.604**	0.408	0.050	0.170	0.489	0.050
q5	**0.653**	0.403	0.088	0.198	0.137	0.134
q6	**0.715**	0.217	0.175	0.162	0.026	0.124
q7	0.188	**0.724**	0.036	0.086	0.224	0.190
q8	0.046	**0.658**	0.214	0.151	0.207	0.214
q9	0.402	**0.552**	0.249	0.419	0.103	0.110
q10	0.381	**0.612**	0.267	0.399	0.334	0.022
q11	0.310	**0.582**	0.357	0.220	0.031	0.129
q12	0.406	0.210	**0.703**	0.041	0.082	0.105
q13	0.416	0.212	**0.696**	0.022	0.133	0.342
q14	0.519	0.016	**0.806**	0.097	0.064	0.048
q15	0.312	0.099	**0.712**	0.299	0.117	0.088
q16	0.049	0.225	**0.409**	0.093	0.319	0.009
q17	0.201	0.309	0.415	**0.609**	0.002	0.064
q18	0.252	0.086	0.306	**0.626**	0.051	0.210
q19	0.307	0.213	0.345	**0.557**	0.299	0.128
q20	0.390	0.299	0.224	**0.712**	0.155	0.288
q21	0.438	0.262	0.103	**0.671**	0.175	0.013
q22	0.069	0.031	0.162	0.168	**0.694**	0.544
q23	0.185	0.214	0.519	0.074	**0.403**	0.354
q24	0.122	0.083	0.509	0.025	**0.68**	0.059
q25	0.334	0.167	0.384	0.107	**0.684**	0.254
q26	0.022	0.211	0.318	0.063	**0.791**	0.401
q27	0.093	0.178	0.203	0.032	0.306	**0.608**
q28	0.329	0.059	0.069	0.319	0.168	**0.405**
q29	0.245	0.216	0.180	0.007	0.250	**0.56**
q30	0.274	0.047	0.023	0.503	0.067	**0.753**
q31	0.095	0.236	0.053	0.448	0.184	**0.758**
Eigenvalues	2.140	1.659	1.564	1.438	1.143	1.075
Present variance explained	21.109	29.948	37.618	44.840	51.742	57.093

a *Rotation converged in 18 iterations*.

### Confirmatory Factor Analysis (CFA)

The validity of the scale was estimated by a confirmatory factor analysis (CFA) utilizing the LISREL 8.80 statistical package. To do so, the following fit indices were examined to evaluate the model fit: the chi-square, which should be non-significant, the chi-square/df ratio, which should be lower than 2 or 3, the root mean square error of approximation (RMSEA), which should be lower than 0.1, the normed fit index (NFI), ranging from 0 to 1(value >0.90 indicating a good fit), the good fit index (GFI) with the cut value >0.90, and comparative fit index (CFI) with the cut value >0.90 (Schreiber et al., 2006). According to Table 4, the chi-square/df ratio (2.364) and the RMSEA (0.057) reached the acceptable fit thresholds (Jöreskog, 1990). The other two fit indices, GFI (0.922), NFI (0.941), and CFI (0.952) were also acceptable.

**Table 4:** Model fit indices.

**Fitting indexes**	****χ**2**	**df**	**χ2/*df***	**RMSEA**	**GFI**	**NFI**	**CFI**
Cut value			<3	<0.1	>0.90	>0.90	>0.90
The first model	990.62	419	2.364	0.057	0.922	0.941	0.952
The second model	696.57	309	2.254	0.055	0.953	0.961	0.971

The result of CFA via two indices of *t*-values and the standardized estimates were reported in Table 5. If the *t*-value (t) is *t* > 1.96 or *t* < −1.96, the result would be 95% statistically significant (Chin, 1998). Also, the standardized coefficient (β) shows the factor loading of each item regarding the corresponding factor. The closer the magnitude to 1.0, the higher the correlation and the greater the factor loading of the item is. A magnitude lower than 0.40 indicates weak factor loading, and the items should be revised or discarded (Chin, 1998).

**Table 5:** The results of confirmatory factor analysis (the first model).

**Constructs**		**Original sample**	**T-statistics**	**Standard error**
Situation selection	q1	0.61	14.27	0.043
	q2	0.36	1.82	0.198
	q3	0.61	10.51	0.058
	q4	0.65	10.02	0.065
	q5	0.72	14.88	0.048
	q6	0.74	14.01	0.053
Situation modification	q7	0.8	17.18	0.047
	q8	0.63	10.63	0.059
	q9	0.63	12.25	0.051
	q10	0.64	14.29	0.045
	q11	0.63	12.23	0.052
Attention deployment	q12	0.68	16.55	0.041
	q13	0.71	16.61	0.043
	q14	0.72	20.34	0.035
	q15	0.77	24.05	0.032
	q16	0.34	1.56	0.218
Reappraisal	q17	0.65	12.36	0.053
	q18	0.68	14.98	0.045
	q19	0.61	12.43	0.049
	q20	0.79	14.34	0.055
	q21	0.7	15.41	0.045
Suppression	q22	0.66	12.23	0.054
	q23	0.31	1.65	0.188
	q24	0.74	14.57	0.051
	q25	0.66	12.96	0.051
	q26	0.74	16.65	0.044
Seeking social support	q27	0.63	13.81	0.046
	q28	0.22	1.12	0.196
	q29	0.64	11.16	0.057
	q30	0.71	13.55	0.052
	q31	0.78	14.73	0.053

As Table 5 indicated, all items had accepted factor loading except items number 2 (*t* = 1.82, β = 0.198), number 16 (*t* = 1.56, β = 0.218), number 23 (*t* = 1.65, β = 0.188), and number 28 (*t* = 1.12, β = 0.196). Therefore, these four items with unacceptable factor loading were omitted from the model.

The second model comprising 27 items on a five-point Likert scale (1 = never, 5 = always) with six components (Appendix II) underwent an identical analysis. As indicated in Table 4, the chi-square/df-ratio (2.254), the RMSEA (0.055), GFI (0.953), NFI (0.961), and CFI (0.971) all reached the acceptable fit thresholds.

**Figure 1:**
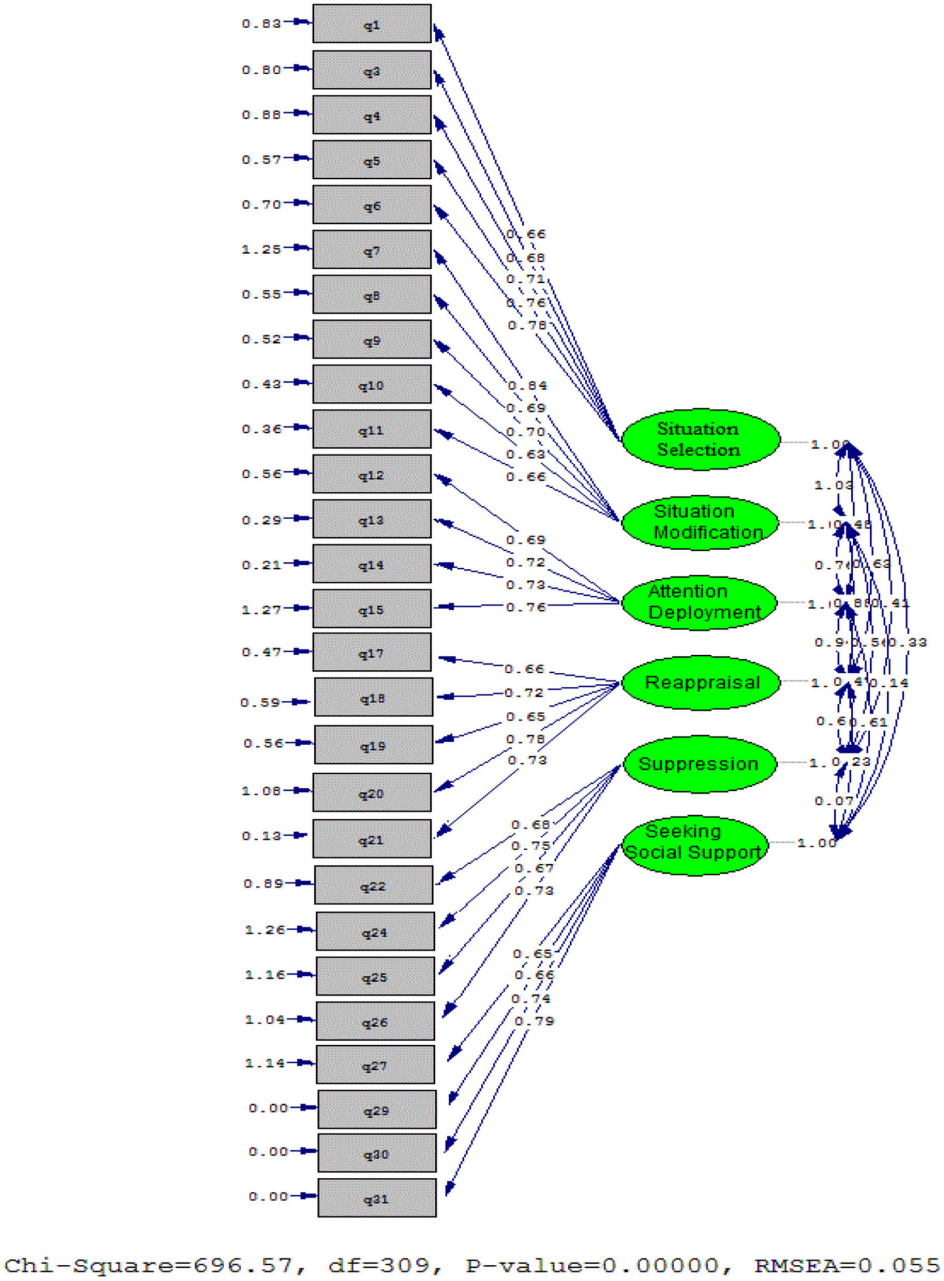
The schematic representation of language teacher emotion regulation and the corresponding items.

The schematic representation of Language Teacher Emotion Regulation and the corresponding items were illustrated in Figure 1. The CFA results (Model 2), Cronbach's alpha, and Average Variance Extracted (AVE) were presented in Table 6.

**Table 6:** The results of confirmatory factor analysis and Cronbach's alpha (the second model).

**Components**		**Original sample**	**T-Statistics**	**Standard error**	**Cronbach's alpha**	**AVE**
Situation selection	q1	0.66	14.78	0.045	0.729	0.518
	q3	0.68	15.73	0.043		
	q4	0.71	17.8	0.040		
	q5	0.76	15.86	0.048		
	q6	0.78	16.27	0.048		
Situation modification	q7	0.84	17.82	0.047	0.718	0.501
	q8	0.69	11.03	0.063		
	q9	0.7	14.37	0.049		
	q10	0.63	12.54	0.050		
	q11	0.66	18.59	0.036		
Attention deployment	q12	0.69	15.32	0.045	0.833	0.526
	q13	0.72	15.81	0.046		
	q14	0.73	16.77	0.044		
	q15	0.76	19.8	0.038		
Reappraisal	q17	0.66	11.78	0.056	0.814	0.504
	q18	0.72	15.99	0.045		
	q19	0.65	11.33	0.057		
	q20	0.78	18.66	0.042		
	q21	0.73	15.41	0.047		
Suppression	q22	0.68	11.69	0.058	0.722	0.502
	q24	0.75	15.31	0.049		
	q25	0.67	10.83	0.062		
	q26	0.73	14.68	0.050		
Seeking social support	q27	0.65	12.68	0.051	0.731	0.507
	q29	0.66	10.31	0.064		
	q30	0.74	14.72	0.050		
	q31	0.79	15.49	0.051		

To check factor loadings of items, β and *t*-values were examined. As Figure 1 and Table 6 demonstrated, all items had accepted factor loadings. Table 6 also displays the reliability of the scale (second model with 27 items) estimated via the Cronbach's alpha. The results of Cronbach's alpha test for all sub-scales of the LTERI (ranging from 0.718 to 0.833) were acceptable. Furthermore, the AVE value for all components of LTERI was higher than 0.5, indicating an acceptable level (Fornell and Larcker, 1981). In the following table, the results of discriminant validity were presented.

To measure discriminant validity, the square root of AVE values should be above the correlation coefficients of each construct with the other constructs (Fornell and Larcker, 1981). The square root of AVE values in Table 7 were presented in bold, which were above the correlation coefficients of each construct with the other constructs. Therefore, discriminant validity for all components of the LTERI was satisfactory.

**Table 7:** The results of discriminant validity.

	**Situation selection**	**Situation modification**	**Attention deployment**	**Reappraisal**	**Suppression**	**Seeking social support**
Situation selection	**0.719**					
Situation modification	0.450^**^	**0.708**				
Attention deployment	0.686^**^	0.405^**^	**0.725**			
Reappraisal	0.467^**^	0.580^**^	0.650^**^	**0.710**		
Suppression	−0.665^**^	−0.508^**^	−0.545^**^	−0.647^**^	**0.708**	
Seeking social support	0.532^**^	0.430^**^	0.559^**^	0.514^**^	0.638^**^	**0.712**

** *Correlation is significant at the 0.01 level (2-tailed)*.

### Cross-Contextual Analysis of Emotion Regulation

The validated scale (LTERI) was employed to determine cross-contextual differences in emotion-regulation strategies between EFL high school and university teachers. As Table 8 presented, the mean scores of teachers in the two educational contexts were different. In situation modification (*M* = 4.476, *SD* = 0.951), attention deployment (*M* = 4.245, *SD* = 0.660), reappraisal (*M* = 4.177, *SD* = 0.884), and seeking social support (*M* = 4.316, *SD* = 0.705), the EFL university teachers got higher mean scores than their counterparts in high schools. Whereas, in situation selection (*M* = 4.298, *SD* = 0.813) and suppression (*M* = 3.792, *SD* = 0.741), the mean scores of EFL high school teachers were more than university teachers. Then, the normality distributions of the data were checked via Kolmogorov-Smirnov Test. Based on the findings, the estimated *p*-value test for all the subscales was >0.05, which indicated that the data had been normally distributed. Therefore, it is implied that parametric methods could be applied. To see whether the observed differences between the groups as mentioned above, were statistically significant an independent-samples *t*-test was applied. In addition, effect size (ES) was employed to estimate the meaningfulness of statistically significant findings. Eta squared was considered to examine the magnitude of the differences. Interpretation of Eta squared is as follows: 0.01 = small effect, 0.06 = moderate effect, and 0.14 large effect (Cohen, 1988).

**Table 8:** Descriptive statistics: emotion regulation sub-scales for EFL high school and university teachers.

**LTERI components**	**Context**	***N***	**Minimum**	**Maximum**	**Mean**	**Std. deviation**
Situation selection	High school teacher	534	1.00	5.00	4.298	0.515
	University teachers	476	1.00	5.00	3.626	0.885
Situation modification	High school teacher	534	1.00	5.00	3.857	0.944
	University teachers	476	2.80	5.00	4.476	0.448
Attention deployment	High school teacher	534	1.00	5.00	3.310	1.052
	University teachers	476	2.40	5.00	4.245	0.682
Reappraisal	High school teacher	534	1.00	4.80	3.183	1.279
	University teachers	476	1.00	5.00	4.177	0.885
Suppression	High school teacher	534	2.00	5.00	3.792	0.809
	University teachers	476	1.00	5.00	3.066	0.809
Seeking social support	High school teacher	534	2.20	5.00	3.510	0.342
	University teachers	476	2.80	5.00	4.316	0.809

As Table 9 summarized, there is a statistically significant difference between EFL high school and university teachers with respect to situation selection (*t* = 14.930, *p* < 0.001, η^2^ = 0.181, large effect), situation modification (*t* = −13.043, *p* < 0.001, η^2^ = 0.144, large effect), attention deployment (*t* = −16.520, *p* < 0.001, η^2^ = 0.213, large effect), reappraisal (*t* = −15.080, *p* < 0.001, η^2^ = 0.184, large effect), suppression (*t* = 13.621, *p* < 0.001, η^2^ = 0.155, large effect), and seeking social support (*t* = −17.335, *p* < 0.001, η^2^ = 0.230, large effect).

**Table 9:** Independent samples *t*-test: high school vs. university teachers regarding their emotion regulation at workplace.

	**Context**	***N***	**Mean**	**Std. deviation**	**Levene's test for equality of variances**		***t-*** **test for equality of means**			**Eta squared**
					**F**	**Sig**.	**t**	**df**	**Sig. (2-tailed)**	
Situation selection	High school teacher	534	4.298	0.813	1.232	0.232	14.930	1,008	0.000	0.181
	University teachers	476	3.626	0.885						
Situation modification	High school teacher	534	3.857	0.944	1.269	0.200	−13.043	1,008	0.000	0.144
	University teachers	476	4.476	0.951						
Attention deployment	High school teacher	534	3.310	0.664	1.159	0.267	−16.520	1,008	0.000	0.213
	University teachers	476	4.245	0.660						
Reappraisal	High school teacher	534	3.183	0.837	2.608	0.112	−15.080	1,008	0.000	0.184
	University teachers	476	4.177	0.884						
Suppression	High school teacher	534	3.792	0.741	0.001	0.978	13.621	1,008	0.000	0.155
	University teachers	476	3.066	0.785						
Seeking social support	High school teacher	534	3.510	0.669	2.417	0.120	−17.335	1,008	0.000	0.230
	University teachers	476	4.316	0.705						

## Discussion

The present study was an attempt to develop a psychometrically sound, valid, and robust inventory to measure language teacher emotion regulation at workplace. For this purpose, the existing literature on emotion regulation, especially teacher emotion regulation, was reviewed. Based on the present though limited literature on the concept of teacher emotion regulation (e.g., Burić et al., 2017; Tsang and Jiang, 2018; Chang, 2020; Richards, 2020; Alipour et al., 2021; Chen and Cheng, 2021), emotion regulation (Gross and Thompson, 2007; Taxer and Gross, 2018) in particular, Gross' process model of emotion regulation (Gross, 1998, 2014), as well as a semi-structured interview with 22 EFL teachers, a model of language teacher emotion regulation was designed. After investigating the content validity of the inventory by a group of experts, exploratory and confirmatory analyses were utilized to examine the construct validity of the proposed six-dimensional model, i.e., situation selection, situation modification, attention deployment, reappraisal, suppression, and seeking social support. The source of the first three dimensions of our proposed model for language teacher emotion regulation (situation selection, situation modification, and attention deployment) is Gross' the process-oriented model of emotion regulation (Gross, 1998). Reappraisal and suppression are originated from Gross and John's study (Gross and John, 2003), and the last dimension in our proposed model, seeking social support, is driven from Jennings and Greenberg (2009) and Taxer and Gross (2018).

EFA examination confirmed all the initial components of the hypothesized model, while CFA did not exhibit statistical support to some items (2, 16, 23, and 28). Furthermore, the calculated model-fit estimates also confirmed the CFA model as a valid measure of language teacher emotion regulation. Item 2 in the first component, situation selection, was discarded in the confirmatory analyses. This can be due to the fact that the emotion regulation strategy suggested in item 2 is near to what is mentioned in item 3. Item 16 (If I feel hopeless at work, I listen to my favorite music or watch my favorite film to forget that) in the third component dealing with attention deployment did not survive the CFA analysis, too. Perhaps, this strategy is not always applicable for every teacher. Listening to music and watching films seems to be among activities that are not very common, especially among middle-aged people. Furthermore, item 23 in the fifth component, suppression, was not confirmed in CFA analysis. It appears that the emotion regulation strategy suggested in item 23 overlapped, item 25 and maybe the reason for not exhibiting statistical support. Among seeking social support, item 28 was discarded. It seems that teachers prefer to share their troubles with their colleagues, experts such as psychologists and school counselors, and close friends but not their relatives as emotion regulation strategies.

In the third phase, the validated scale was employed in two different milieus for language teaching and learning in Iran to determine the cross-contextual discrepancies in English language teachers' emotion regulation strategies. To date, no known studies have reported specifically a cross-cultural analysis of emotion regulation strategies among EFL high school and university teachers. Leafing through the existing literature indicates that studies on language teacher emotion regulation have not been brought to the foreground of research foci. This scattered literature may be due to the limited attention to language teacher emotion regulation (Richards, 2020; Alipour et al., 2021; Fathi et al., 2021) and the absence of psychometrically sound instruments on language teacher emotion regulation at workplace.

The findings of the current investigation showed that the strategies that EFL high school teachers employed in emotion regulation at workplace are different from EFL university teachers. According to the results, university teachers are more successful in emotion regulation at workplace than EFL high school teachers. Among the components of LTERI, EFL university teachers tend to deploy situation modification, attention deployment, reappraisal, and seeking social support. While EFL high school teachers prefer situation selection and suppression. This finding can be justified with reference to the fact that these two educational contexts have different English teaching and learning objectives, teaching methods, and procedures, as well as teacher and learner roles which may affect their experienced emotions and consequently their employed emotion regulation strategies. This is consistent with previous experimental surveys, though limited in L2 settings and quite rare among Iranian EFL settings (Hargreaves, 2001; Kunter et al., 2011; Richards, 2020; Fathi et al., 2021).

Moreover, EFL university teachers with higher education appreciate emotion regulation strategies rooted more in self-regulation, self-awareness, reasoning, and higher-order thinking skills (Morris and King, 2020; Alipour et al., 2021; Heydarnejad et al., 2021). EFL high school teachers, on the other hand, mostly try to avoid challenging emotional situations or suppress their experienced emotions. This can be due to the context of schools that do not provide developmental programs targeting emotion regulation. Also, high school teachers are exposed to more rigid rules in some countries like Iran that may cause more emotional exhaustion, burn out, and suppression (Akbari et al., 2020; Angelica and Katz, 2020). Taken together, the yielded results of the present study led to this conclusion that language teachers' emotions regulation in all educational contexts (school, private institute, and university) is critical in the process of effective teaching and educational psychology.

## Conclusion

As yet, language teacher emotion regulation was not explored quantitatively, and this knowledge gap may be due to a lack of psychometrically and conceptually sound self-report instruments. The development and validation of the Language Teacher Emotion Regulation Inventory (LTERI) may foster future studies in this domain, which is the aim of the researchers of the present study. Adding the implications of the current research in pre-service and in-service teacher training programs can pave the way for triggering self-aid skills, which are of great help, especially in the global crisis of the Covid-19 pandemic. It is also recommended to policymakers, curriculum designer, and material developers to design and provide educational materials which focus on practicing self-awareness, self-reflection, and self-evaluation to guarantee the well-being of the society.

The results of the present study need to be interpreted in light of the following limitations. First, since the results of this study provided initial evidence of the validity of LTERI as a language teacher emotion regulation in Iranian context, further research is needed to capture the predictive and consequential validity of the scale. Second, further studies such as class observations and focused-grouped interviews are suggested to evaluate the outcomes of the present study. Third, future investigations can take more mixed-method approaches to inspect the interplay among language teacher emotion regulation and a number of other teacher-related variables such as motivation, burnout, reflective teaching, critical thinking, teaching style, autonomy, and immunity. Fourth, while LTERI has been administered to Iranian samples of teachers, it is highly recommended to use this instrument across different countries and among other samples of language teachers. Finally, further research should be undertaken to investigate whether language teacher emotion regulation affects language learners' emotion regulation.

## Data Availability Statement

The raw data supporting the conclusions of this article will be made available by the authors, without undue reservation.

## Ethics Statement

The studies involving human participants were reviewed and approved by Hakim Sabzevary University. Written informed consent for participation was not required for this study in accordance with the national legislation and the institutional requirements.

## Author Contributions

TH, GZ, SG, and SA contributed to conception and design of the study and wrote the sections of the manuscript. TH organized the database, performed the statistical analysis, and wrote the first draft of the manuscript. All authors contributed to manuscript revision, read, and approved the submitted version.

## Conflict of Interest

The authors declare that the research was conducted in the absence of any commercial or financial relationships that could be construed as a potential conflict of interest.

## Publisher's Note

All claims expressed in this article are solely those of the authors and do not necessarily represent those of their affiliated organizations, or those of the publisher, the editors and the reviewers. Any product that may be evaluated in this article, or claim that may be made by its manufacturer, is not guaranteed or endorsed by the publisher.

## Appendix I

### Interview Questions

1. What are the contextual triggers of emotion regulation for you as a language teacher at workplace?

2. How do such emotion regulation strategies affect your behavior?

3. What kinds of emotion regulation strategies do you apply to manage the experienced emotions at workplace?

4. What kinds of emotion regulation strategies do you use to modify the experienced emotions at workplace?

5. What kinds of emotion regulation strategies do you employ to distract your attention from the experienced emotions at workplace?

6. At the moment of emotional experiences at workplace how do you reappraise different emotions?

7. On what occasion do you suppress your emotions at workplace?

8. How can social support affect the management of your emotional experiences at workplace?

## Appendix II

### Language Teacher Emotion Regulation Inventory (LTERI)

**Table:**

**Language teachers' emotion regulation strategies**		**Items**
A. Situation selection	1	In my classroom, I avoid conflicting or emotionally annoying situations.
	2	I try to evade unpleasant discussions.
	3	I avoid conflicting or emotionally disturbing situations in the staff room.
	4	I try to avoid discussing with troublesome parents.
	5	In my work, I try to avoid a certain situation that may bring about undesirable emotions.
B. Situation modification	6	When I feel helpless at work, I think about my teaching methods critically.
	7	If my students make me angry in language classes, I try to advise them.
	8	When an unpleasant discussion is raised in my classes, I try to change the topic.
	9	As I improve my knowledge and skills, I can react better in stressful situations at work.
	10	When I face an upsetting conversational topic, I try to substitute it with suitable ones.
C. Attention deployment	11	When I feel anxious in my language classes, I shift my attention to something pleasant.
	12	If I feel frustrated in language classes, I try to engage myself in different class activities to forget it.
	13	In language classes, if I feel unhappy, I try to think about something interesting.
	14	When I feel upset in my language classes, I redirect my attention to more pleasant matters.
D. Reappraisal	15	I try to reduce the tension experienced in my language classes by reminding myself that there are more important things in my life.
	16	If my students' misbehavior makes me angry, I remind myself that they are inexperienced.
	17	When I feel ashamed, I remind myself that I can do better in the future.
	18	If for some reasons, I feel upset at work, I remind myself of my goals in my life.
	19	If I feel hopeless at work, I calm myself by viewing things from another perspective.
E. Suppression	20	If I feel anxious in my language classes, I try to suppress that.
	21	If I feel helpless in my language classes, I disregard that.
	22	If for some reasons, I feel angry in my language classes, I overlook that.
	23	When I feel unhappy at work, I ignore that.
F. Seeking social support	24	When I feel frustrated in my language classes, I share my troubles with my colleagues.
	25	When I feel hopeless in my language classes, I seek advice from experts such as psychologists and school counselors.
	26	If I feel nervous in my language classes, I talk about it with someone who can understand me.
	27	To get my mind off an upsetting situation at work, I talk about it with someone who is close to me.
